# Safety and Efficacy of Intravitreal Chemotherapy (Melphalan) to Treat Vitreous Seeds in Retinoblastoma

**DOI:** 10.3389/fphar.2021.696787

**Published:** 2021-07-12

**Authors:** Yacoub A. Yousef, Mays Al Jboor, Mona Mohammad, Mustafa Mehyar, Mario D. Toro, Rashed Nazzal, Qusai H. Alzureikat, Magdalena Rejdak, Mutasem Elfalah, Iyad Sultan, Robert Rejdak, Maysa Al-Hussaini, Ibrahim Al-Nawaiseh

**Affiliations:** ^1^ Department of Surgery, Ophthalmology Division, King Hussein Cancer Center, Amman, Jordan; ^2^ The Eye Speciality Hospital, Amman, Jordan; ^3^ Chair and Department of General and Pediatric Ophthalmology, Medical University of Lublin, Lublin, Poland; ^4^ Department of Ophthalmology, University of Zurich, Zurich, Switzerland; ^5^ Medical Faculty of Warsaw, Warsaw, Poland; ^6^ Department of Special Surgery, Faculty of Medicine, The University of Jordan, Amman, Jordan; ^7^ Departments of Pediatrics Oncology, King Hussein Cancer Center, Amman, Jordan; ^8^ Departments of Pathology, King Hussein Cancer Center, Amman, Jordan

**Keywords:** chemotherapy, melphalan, ocular toxicity, retinoblastoma, vitreous seeds

## Abstract

**Background:** Active vitreous seeds in eyes with retinoblastoma (Rb) adversely affects the treatment outcome. This study aimed to investigate the safety and efficacy of intravitreal melphalan chemotherapy (IViC) as a treatment for recurrent and refractory vitreous seeds in patients with Rb.

**Methods:** We used a retrospective non-comparative study of patients with intraocular Rb who had vitreous seeds and were treated by IViC (20–30 μg of melphalan) using the safety-enhanced anti-reflux technique. Tumor response, ocular toxicity, demographics, clinical features, and survival were analyzed.

**Results:** In total, 27 eyes were treated with 108 injections for recurrent (16 eyes) or refractory (11 eyes) vitreous seeds after failed systemic chemotherapy. A total of 15 (56%) were males, and 20 (74%) had bilateral disease. At diagnosis, the majority (*n* = 21) of the injected eyes were group D, and *n* = 6 were group C. Vitreous seeds showed complete regression in 21 (78%) eyes; 100% (*n* = 10) for eyes with focal seeds; 65% (*n* = 11/17 eyes) for eyes with diffuse seeds (*p* = 0.04); 7 (64%) eyes with refractory seeds; and 14 (87%) eyes with recurrent seeds showed complete response (*p* = 0.37). In total, 16 (59%) eyes developed side effects: retinal toxicity (48%), pupillary synechiae (15%), cataracts (30%), iris atrophy (7%), and retinal and optic atrophy (4%). Only one child was lost to follow-up whose family refused enucleation and none developed orbital tumor recurrence or distant metastasis.

**Conclusion:** IViC with melphalan is effective (more for focal than diffuse seeding) and a relatively safe treatment modality for Rb that can improve the outcomes of eye salvage procedures. However, unexpected toxicity can occur even with the standard dose of 20–30 μg.

## Introduction

Retinoblastoma is the most prevalent pediatric intraocular tumor (Kivela, 2009) for which enucleation is the ultimate treatment. Eye (globe) salvage can be achieved in many cases by employing a variety of management modalities, which includes systemic and/or regional chemotherapy and consolidation therapy (thermal and cryotherapy). However, managing recurrent or persistent vitreous seeding has been a major impediment that has reduced the apparent eye salvage rates in patients with advanced intraocular disease (Chan et al., 1995; Shields et al., 2002; Yousef et al., 2020c; Yousef et al., 2021).

Early studies have reported that the salvage rates for the groups A, B, and C retinoblastoma (classified using the International Intraocular Retinoblastoma Classification, IIRC) (Linnmurphree, 2005) were generally high (81–100%) compared to only about 50% for group D eyes (Shields et al., 2006; Yousef et al., 2021; Amine et al., 2021). This unfavorable outcome for in group D may be attributed to the presence of massive vitreous and/or subretinal seeds. Eventually, with the introduction of selective ophthalmic artery chemotherapy (IAC), the eye globe salvage rate for D eyes increased to 70%, while only 64% of D eyes with massive vitreous seeds could be managed with IAC (Munier et al., 2011; Abramson et al., 2012). Thus, radiotherapy (external beam radiation therapy, EBRT) continued to be used to treat the few cases of recurrent vitreous seeds to attain an improved tumor control rate of 46–91% (Shields et al., 2009; Yousef et al., 2020b) and avoid enucleation. Unfortunately, this was associated with a higher risk of secondary primary malignancies (Kleinerman et al., 2005).

Subsequently, intra-vitreal chemotherapy (IViC), specifically with melphalan, emerged in 2012 as a promising treatment technique for active recurrent or persistent vitreous seeds, improving the eye salvage rate to about 87% in an initial report (Munier et al., 2012a). The authors also reported an 81% tumor control in the eyes with active vitreous seeds that were initially planned for enucleation (Munier et al., 2012a). Most reports on the safety of intraocular melphalan have documented minimal ocular toxicity after an injected dose of 20–30 μg (calculated based on the patient’s age) in the Caucasian populations of Europe and America (Munier, 2014; Shields et al., 2014; Francis et al., 2015). On the other hand, the reported toxicity was higher in Chinese retinoblastoma patients (Xue et al., 2019).

Therefore, the present study aimed to further investigate both the safety and toxicity of IViC with melphalan to treat eyes with intraocular Rb presenting with refractory vitreous seeds or recurrent seeds after failed systemic intravenous chemotherapy combined with focal consolidation in a lower-middle-income country in the Middle East (Jordan).

## Patients and Methods

This is a retrospective non-comparative analysis approved by the Institutional Review Board at the King Hussein Cancer Center (Amman, Jordan) (18KHCC27). The review board waived the need to obtain consent owing to the retrospective nature of the study. The study included 27 eyes with intraocular Rb (from 27 patients) with refractory or recurrent vitreous seeds who received the IViC treatment between January 2014 and June 2020. Eligibility criteria for administering IViC were (Munier et al., 2012b; Moulin et al., 2012):

(1) No tumor invasion of the anterior or posterior chamber.
(2) No associated retinal or anterior hyaloid detachment.
(3) Presence of an entry site for the injection that is free of active tumor or active vitreous seeds.

All group E and D eyes with massive vitreous seeds in all four quadrants of the eye where no clear safe quadrant for injection was available were not eligible to receive this treatment and were excluded. All eligible patients were examined by an ocular oncologist under anesthesia and fundus dilated to thoroughly examine the retina, and the retinal photos were documented using Retcam II (Clarity Medical Systems, CA, United States). Tumor location, seeds’ features, and response to treatment were documented adequately. Informed consent for the treatment was obtained from the patients’ parents, following which the procedures were performed under completely sterile conditions in the operating room.

### Definitions

Vitreous seeding was classified into focal vitreous seeds (seeds limited to only one quadrant) and diffuse vitreous seeds (seeds that were extensive and detected in more than one quadrant of the eye globe). Based on the distance of the vitreous seeds from the retinal surface, they were grouped as either less than 3 mm or more than 3 mm from the surface of the retina.

**FIGURE 1:**
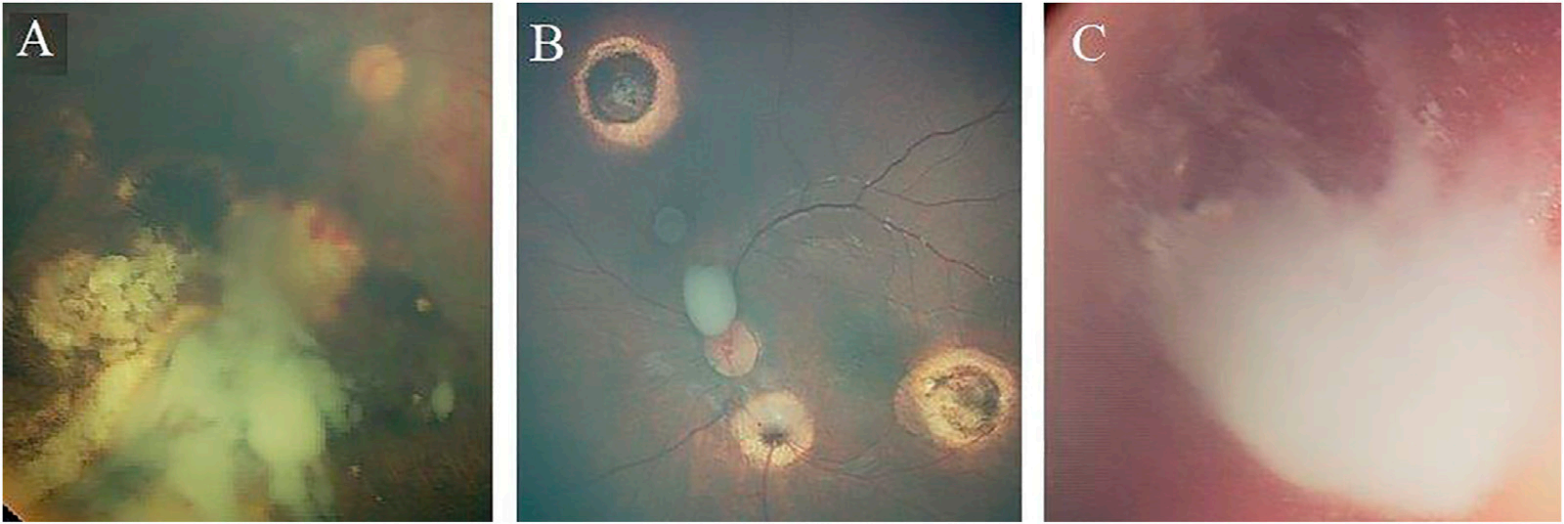
Morphologic types of recurrent and refractory vitreous seeds in Rb patients. **(A)** Eye that harboures massive cloud (type III vitreous seeds). **(B)** Eye that harbours mixture of 2 types of vitreous seeds; sphere (type II vitreous seeds), and cloud (type III vitreous seeds). **(C)** Large cloud (type III vitreous seeds) associated with massive Dust (type I vitreous seeds)..

**FIGURE 2:**
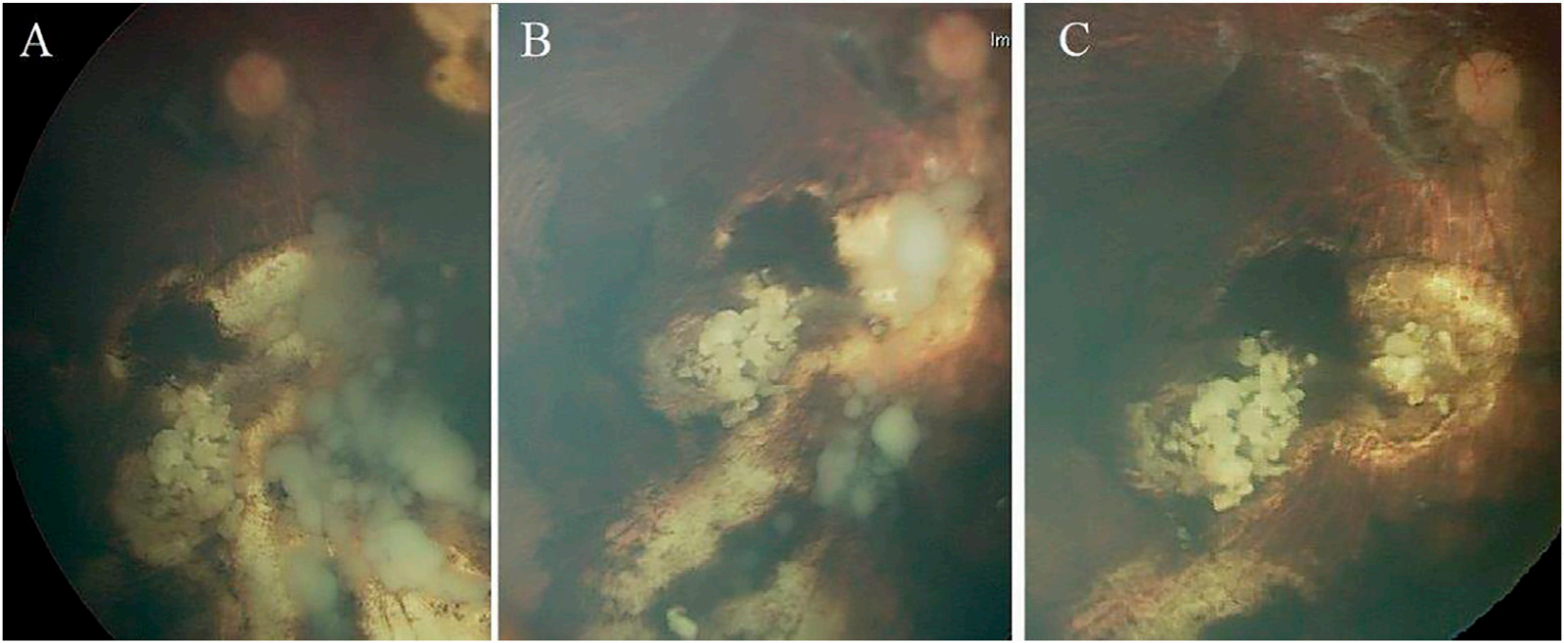
Treatment response of vitreous seeding to intravitreal melphalan injection; **(A)** massive cloud of vitreous seeds that regressed partially after 3 injections **(B)** and totally disappeared after total of 6 injections **(C)**. This shows type 0 pattern of regression where vitreous seeds disappeared completely.

Further, based on the pattern of vitreous seeding, three subtypes were defined: Type I (dust-like seeds), Type II (sphere-like seeds), and Type III (clouds-like seeds) (Susskind et al., 2016) (Figure 1). Response of vitreous seeds to the IViC was categorized into 3 patterns: Type 0 (complete disappearance of the vitreous seeds), Type I (calcific vitreous seeds), and Type II (amorphous vitreous seeds) (Figure 2).

### Surgical Technique

First, the injection site was examined by indirect ophthalmoscopy followed by Ultrasound Bio-Microscopy (UBM) to confirm a clear site for injection (no tumors or retinal detachment). Ocular hypotony was induced by withdrawing 0.05–0.1 ml of aqueous fluid, which was sent for cytopathology screening. A 30-gauge sterile needle was used to inject the chemotherapy drug, melphalan, in the specified amount based on the patient’s age. All patients received 20–30 μg of melphalan intravitreally by inserting the needle in pars plana 2.5–3.5 mm away from the limbus, perpendicular to the sclera toward the vitreous humor and away from the anatomical location of the lens.

Cryotherapy was applied (triple-freeze-thaw cryotherapy) at the injection site immediately afterward to sterilize the needle track from any possible active tumor cells. The eye was shaken manually after each injection to spread the chemotherapy in the vitreous. Each patient received a minimum of three injections and a maximum of eight injections 1–2 weeks apart.

### Drug Preparation

Commercially available 50 mg lyophilized powder of melphalan hydrochloride was reconstituted with 0.9% sodium chloride solution (preservative-free). Initially, a concentration of 5 mg/ml of melphalan was achieved by adding 10 ml of 0.9% normal saline followed by vigorous shaking of the drug till the solution becomes clear. A 0.2 mg/ml (200 μg/ml) sterile solution was obtained by mixing 1 ml of melphalan with 24 ml 0.9% sodium chloride. Then, the reconstituted drug (0.3 ml melphalan) was transferred to a 1 ml lock syringe through a 5 μ filter. The dosage is adjusted and customized accordingly; 20 μg/0.10 ml for 0–12 months of age, 25 μg/0.125 ml for 1–3 years of age, and 30 μg/0.15 ml for patients aging 3 years and above (Manjandavida and Shields, 2015). All associated active retinal tumors were treated by focal consolidation therapy (cryotherapy, transpupillary thermotherapy, or radioactive plaque therapy) as needed.

### Outcome Measurement

Response to treatment was evaluated by examination under anesthesia after each injection and before the next injection. Successful therapy was defined by avoidance of enucleation or EBRT. Good tumor response was defined as a complete response (regression of all active seeds and no recurrence detected at 6 months after the last injection), while failed treatment was defined as residual active vitreous seeds and/or recurrent vitreous seeds within 6 months from the last injection.

Retinal toxicity was graded based on Munier’s report (Munier, 2014) into five grades: Grade I: less than 2 clock hours of salt-and-pepper retinopathy in the peripheral retina and anterior to or at the equator; Grade II: greater than 2 clock hours of retinopathy that extends anteriorly or at the level of the equator; Grade III: retinopathy that extends posterior to the equator but not involving the macula; Grade IV: retinopathy involving the macula (maculopathy); and Grade V: extensive pan-retinopathy with concomitant optic disc atrophy.

Fisher’s exact test was used to determine statistical significance and a *p*-value of <0.05 was considered statistically significant.

## Results

A total of 108 IViC injections were administered in 27 eyes from 27 patients with recurrent or refractory vitreous seeds (mean = median = 4 injections per eye; range = 3–8 injections). A standardized dose of 20–30 μg melphalan was given to all patients based on their age.

### Demographics and Clinical Features

Of the 27 patients, 15 (56%) were boys, 12 (44%) were girls, and most of them (*n* = 20; 74%) had bilateral Rb. In total, 11 (41%) eyes had persistent refractory seeds, and 16 (59%) eyes had recurrent active seeds. Notably, only three eyes had recurrent seeds within 6 months after treatment with I-125 radioactive plaque therapy. At the time of diagnosis, the mean age was 17 months (median = 13, range = 4–50 months), and 6 eyes (22%) belonged to group C and 21 (78%) eyes to group D (Table 1). Other features of the treated vitreous seeds are summarized in Table 1.

**TABLE 1:** Demographics, tumor characteristics, and management outcome.

Feature		No.	Complete response		Failure		*p* Value
Total	27 Patients	27	21	78%	6	22%	
Gender	Female	12	9	75%	3	25%	0.62
	Male	15	12	80%	3	20%	
Laterality	Unilateral	7	6	86%	1	14%	1.00
	Bilateral	20	15	75%	5	25%	
Vitreous seeds status	Persistent	11	7	64%	4	36%	0.37
	Recurrent	16	14	87%	2	13%	
IIRC	Group C	6	5	83%	1	17%	1.00
	Group D	21	16	76%	5	24%	
Associated subretinal seeds	With SRS	17	14	82%	3	18%	0.32
	Without SRS	10	7	70%	3	30%	
Tumor location	Macular	13	10	77%	3	33%	0.64
	Extramacular	14	11	79%	3	21%	
Type of vitreous seeds	Type I dust	8	6	75%	2	25%	0.61
	Type II sphere	3	3	100%	0	0%	
	Type III clouds	12	9	75%	3	25%	
	Mixed	4	3	75%	1	25%	
Distance from retina	<3 mm	7	6	86%	1	14%	1.00
	>3 mm	20	15	75%	5	25%	
Severity of vitreous seeds	Diffuse	17	11	65%	6	35%	0.04
	Focal	10	10	100%	0	0%	

Most of the eyes had diffuse vitreous seeds (*n* = 17, 63%), and n = 10 (37%) eyes had focal vitreous seeds. The distribution of the pattern of vitreous seeding in the treated eyes was: Type III clouds in 12 (44%) eyes, Type I dust in 8 (30%) eyes, Type II sphere in 3 (11%) eyes, and mixed in 4 (15%) eyes.

### Previous Treatments

Primary systemic intravenous chemotherapy was given to all patients in this series (6–8 cycles of carboplatin + vincristine + etoposide; CVE). One patient received additional three cycles of topotecan systemic chemotherapy after the CVE.

The 6 group-C eyes and 13/21 group-D eyes received six cycles CVE, the 8 of group D eyes received eight cycles of CVE, and one of them received additional three cycles of systemic Topotecan. All affected eyes were also treated with focal consolidation therapy (transpupillary thermotherapy or cryotherapy as needed).

Three eyes were previously treated by radioactive Iodine-125 plaque therapy before receiving IViC. All these eyes had massive vitreous seeds more than 2 mm from the surface of the active tumor at time of radioactive plaque therapy. They showed initial regression or at least no progression immediately after the plaque therapy, and presented with vitreous seeds progression within 6 months from the date of radioactive plaque, while the main tumor was still inactive. Four eyes received periocular (sub-conjunctival) carboplatin injections 3 months before the IViC treatment. All the eyes that received radioactive plaque or subconjunctival carboplatin were group D eyes.

### Response to Intravitreal Chemotherapy

Out of the 27 injected eyes, 21 (78%) eyes showed a complete response with no active vitreous seeds at the last day of follow-up (median number of injections = 4; range = 3**–**8). Complete suppression (type 0 response) was seen in *n* = 14 (52%) eyes, calcific seeds (type I response) in *n* = 8 (30%) eyes, and amorphous seeds (type II response) in *n* = 5 (19%) eyes. In total, five out of the 6 group-C eyes (83%) and 16 out of the 21 group-D eyes (76%) showed a complete response (*p* = 1.0). A complete response was noticed in 7 out of the 11 eyes with persistent vitreous seeds (64%) and in 14 out of 16 eyes with recurrent vitreous seeds (88%), which was not statistically significant (*p* = 0.37) (Table 1).

The treated vitreous seeds were successfully controlled by IViC in 14 (82%) eyes that had active sub-retinal seeds at the time of injection and in 7 (70%) eyes that had no active or recurrent sub-retinal seeds (*p* = 0.32). Seeding in 6 (86%) of the treated eyes where the seeds were closer than 3 mm to the retina and 15 (75%) of the eyes where the seeds were more than 3 mm far from the retina was completely controlled by IViC (*p* = 1.00). On the other hand, all (100%) eyes with focal vitreous seeds were controlled by IViC, while only 11 (65%) of the eyes with diffuse vitreous seeds were controlled (*p* = 0.04). The number (median) of IViC injections mandated for treatment of the active seeds was three injections for eyes with dust-like seeds, four injections for eyes with sphere-like seeds and mixed seeds, and 5 for clouds vitreous seeds (Table 2).

**TABLE 2:** Correlation between tumor characteristics and the number of injections.

Feature		No.	Complete response		Number of injections (Median)
Total	27 patients	27	21	78%	4
Number of injections	Total 108 injections (mean and median, 4 and 4 injections per eye; range, 3**–**8)				
Vitreous seeds status	Persistent	11	7	64%	5
	Recurrent	16	14	87%	3
Type of vitreous seeds	Type I dust	8	6	75%	3
	Type II sphere	3	3	100%	4
	Type III clouds	12	9	75%	5
	Mixed	4	3	75%	4
Severity of vitreous seeds	Diffuse	17	11	65%	5
	Focal	10	10	100%	3

### Management Outcome and Complications

After a median follow-up of 42 months after the last IViC injection (range 9–72 months), 6 (22%) eyes failed the treatment and the patient had to undergo enucleation or EBRT.

Two eyes had massive recurrent vitreous seeds involving more than one quadrant (4 and 6 months after the last injection), one eye had ciliary body and anterior chamber invasion, and one eye had phthisis. Four of these were enucleated; one eye presented with massive recurrent vitreous seeds with concomitant active massive sub-retinal seeds 9 months after the IViC injections. The patient had a single eye, so he received three more cycles of systemic chemotherapy, and thereafter ended with EBRT. The sixth patient had a recurrent retinal tumor, sub-retinal seeds, and vitreous seeds associated with dense cataract, and a decision for enucleation was taken. The family refused this decision and decided not to treat. After getting lost in follow-up, they came back with a recurring orbital tumor. Even though parents refused further management and lost for follow-up again.

Out of 27 treated eyes, treatment side effects were seen in 16 (59%) eyes. In total, 13 (48%) eyes developed retinal toxicity: seven eyes had Grade I toxicity, three eyes had Grade II, two eyes had Grade III toxicity, no eye had Grade IV toxicity, and one eye developed Grade V toxicity (pan-retinopathy with optic atrophy). No patient developed endophthalmitis. Cataract was seen in eight (30%) eyes, five (19%) of them had a dense cataract that affected the fundus view, and, interestingly, three of them were previously treated by radioactive plaque therapy. All patients who developed significant cataracts (*n* = 5) had their cataract extracted surgically (with intraocular lens implantation without perforation of the posterior capsule) 12 months after the last injection, all of which were stable with no tumor or seeds recurrence after the surgery. Other complications included four (15%) eyes with pupillary synechia, two (7%) eyes with iris atrophy, 1 (4%) eye with optic atrophy, one (4%) eye with phthisis bulbi, and one (4%) eye with a retinal hemorrhage (Table 3).

**TABLE 3:** Side effects of intravitreal melphalan chemotherapy.

	Number of eyes	%
Median follow up (total 27 eyes)	42 months (range 6–72 months)	
Total number with eyes with side effects	16 eyes	59
Tumor recurrence^a^	6 eyes	22
Median time for recurrence	6 months (range 3–12 months)	
Retinal toxicity^b^	13 eyes	48
Pupillary synechia	4 eyes	15
Iris atrophy	2 eyes	7
Optic atrophy	1 eye	4
Cataract (Dense)^c^	8 (5) eyes	30 (19)
Hypotonia and phthisis bulbi	1 eye	4
Retinal hemorrhages	1 eye	4
Endophthalmitis	None	0
Orbital tumor recurrence	1^d^	4
Distant metastasis	None	0

a Six eyes showed recurrent active tumor; three had massive recurrent vitreous seeds involving more than one quadrant (3, 4, and 6 months after the last injection), one eye had ciliary body and anterior chamber invasion, one eye had recurrent subretinal and vitreous seeds, and one eye had phthisis.

b In total, 13 (48%) eyes developed retinal toxicity; seven eyes had Grade I toxicity, three eyes had Grade II toxicity, two eyes had Grade III toxicity, none had Grade IV toxicity, and one eye had Grade V toxicity (pan-retinopathy with optic atrophy)

c Three of these eyes had radioactive plaque therapy.

d One of the patients had a recurrent retinal tumor, subretinal seeds, and vitreous seeds associated with dense cataract, and the decision was for enucleation. The family refused this decision and decided not to treat. After getting lost in follow-up, they came back with orbital tumor recurrence.

All patients were alive by the last day of follow-up. Moreover, other than the child whose family refused enucleation and was lost in follow-up, no child in this study had a recurrence of orbital tumor, and none had metastasis to the CNS or the bone marrow. The eyes that had normal macula and optic disc had a median vision of 0.5 (range = 0.2–0.8).

## Discussion

In this study, 78% of the eyes with intraocular Rb that harbored active refractory or recurrent vitreous seeds benefitted from intravitreal melphalan chemotherapy and avoided enucleation and EBRT. However, 59% of the eyes showed some kind of complications ranging from mild retinal pigmentation to severe retinal toxicity and atrophy.

For decades, ophthalmologists have avoided inserting a needle inside an eye with active Rb (for both diagnosis and treatment) due to the potentially higher risk of tumor dissemination through the site of injection. Most of the published data about intravitreal chemotherapy for Rb belongs to developed countries where quality control is mandatory. This study was conducted in Jordan (a developing country) and we followed a strict safety-enhanced anti-reflux protocol (Munier et al., 2012a) to prevent tumor dissemination *via* the route of injection. This protocol included injection of the chemotherapeutic drug in the pars plana in the tumor-free quadrant, lowering the intraocular pressure, and freezing the needle track (triple-freeze-thaw) immediately after each injection. Because of this protocol, other than one child who refused treatment and was lost in follow-up, no child developed extraocular tumor dissemination or presented with tumor metastasis over the median 42 months (9–72 months) follow-up period. This safety data is supported by the data from the first report about the safety of anti-reflux technique for IViC by Munier et al. who were the first to elucidate this protocol in treating 23 eyes with Rb in Switzerland (Munier et al., 2012a), and they too did not report any case of metastasis (in a follow-up period of 22 months). Similarly, subsequent reports with a slightly longer follow up (not more than 66 months) that followed similar inclusion criteria and used the same injection protocol did not report any case of extraocular invasion or distant metastasis (Munier, 2014; Berry et al., 2017; Kiratli et al., 2017; Xue et al., 2019).

It is noteworthy that we followed our patients for a longer period, and no orbital recurrence or distant metastases were encountered. This indicates that IViC can be applied safely for Rb patients all around the world as long as the treating team followed a strict injection protocol. On the other hand, other studies about eyes that received intravitreal chemotherapy who have not mentioned clear selection criteria and did not strictly follow the anti-reflux measures reported a 0.4% chance of post-operative orbital tumor invasion and a 4.4% chance of brain metastasis, which is a significant risk for these children (Kaneko and Suzuki, 2003). This difference in the incidence of orbital recurrence and metastasis highlights the importance of following the eligibility criteria and strict anti-reflux injection technique to prevent metastasis. In our series, we saved 78% of the eyes with vitreous seeds that were otherwise planned to be enucleated, which is comparable to the previously reported data of 79–100% salvage rates with a dose of 20–30 μg, and 68–83% salvage with a dose of 8–20 μg (Ghassemi and Shields, 2012; Munier et al., 2012a; Shields et al., 2014; Xunda et al., 2016; Berry et al., 2017).

Earlier reports have shown melphalan to be the single most effective chemotherapy agent against Rb and being less toxic if used at specific doses (Inomata and Kaneko, 2004). This was based on previous *in vitro* studies by Inomata and Kaneko (1987), who found this drug to be the most efficient among the 12 tested. Preclinical studies in the rabbit have established that the vitreous concentration necessary (5.9 μg/ml) for tumor control can be achieved without retinal toxicity (Ueda et al., 1995). When extrapolated to the human vitreous volume, the injected dose corresponds to the injection of 20–30 μg. The possible side effects of IViC treatment include cataracts, uveitis, endophthalmitis, retinal toxicity, vitreous hemorrhage, optic atrophy, extraocular tumor extension, and metastasis. Most of the published data about local toxicity of IViC are from the Caucasian populations and very few for the Mediterranean and South-East Asia populations. Smith et al., 2013 presented a correlation between the dose of melphalan and the risk of ocular toxicity and showed that a 30 μg dose has fewer side effects than higher doses. Further, Francis reported a higher rate of ocular toxicity in more deeply pigmented dark eyes (Francis et al., 2014; Susskind et al., 2016; Francis et al., 2017) as the pigmentation may absorb higher levels of chemotherapy (like melphalan) leading to a higher retinal, RPE, and choroidal toxicity. Our Jordanian population falls in this group of pigmented eyes, so we expect higher rates of ocular toxicity than the Caucasian population.

Francis also reported anterior segment toxicity in 7% of eyes after IViC injections in the Caucasian population, while in China, 43% of patients developed pupillary synechiae, 40% had iris atrophy, and 27% developed cataracts (Francis et al., 2017; Xue et al., 2019). Chao et al. reported a case of diffuse chorioretinal atrophy after injecting a single dose of 8 μg melphalan (Chao et al., 2016). In our study, pupillary synechiae and iris atrophy occurred in 15% and 7% of patients, which is less than the reported value for China, but 30% of the patients developed cataracts, which is still higher than the Chinese numbers. Cataract in our patients could be attributed to the intravitreal melphalan injection, although three of these patients had previously undergone radioactive plaque therapy, while the other five eyes did not receive any form of radiation therapy. Overall, we are unable to conclude whether these anterior segment side effects are caused by IViC as almost all the eyes in our and other studies received more than two or three treatment modalities, including intravenous chemotherapy, IAC, cryotherapy, and laser therapy. Similarly, the high rate of cataract cannot be correlated to the close distance between the tip of the needle (and the injected melphalan) and the posterior surface of the lens, as all eyes received injections by the same technique, and all eyes were vigoureously shaked immediately after each injection. Strikingly, in Japan (Kaneko and Suzuki, 2003), the reported visual outcome for eyes that had extra macular tumors and were treated by 8–20 μg melphalan (lower dose than other studies) was ≥ 0.5 in 27% of the injected eyes, a result which is comparable to the visual outcome in our patients who were given a higher dose of 20–30 μg. This suggests that a dose of 20–30 μg melphalan given intravitreally is safe and effective for active vitreous seeds. Furthermore, it could be useful develop a biodegradable deliver system to inject melphalan in order to improve the pharmacology profile and the safety profile (Conti et al., 1997).

Because of emergence of cases that are resistant to melphalan alone, Ghassemi et al. (2014) reported a total of 17 combined sessions of intravitreal injections using combined melphalan and topotecan, and they achieved complete response of vitreous seeds in 100% of eyes with minimal toxic effects, with a median of two injections (mean, 1.9). Similarly Kiratli et al. showed that the combined use of intravitreal melphalan and topotecan provide better results in terms of avoiding enucleation and vitreal and subretinal seed control, as the enucleation rate was 62% for eyes that received melphalan alone, while wnucleation rate was 11% in eyes that received combination of intravitreal melphalan and topotecan (Kiratli et al., 2020). In our study, all eyes received melphalane a lone, and the eye salvage rate was 78% that is notably less than for eyes received combined melphalan and topotecan, therefore administration of melphalan and topotecan combination may have favorable response over melphalan alone, and it mandates lower number of injections to control the seeds, but more studies with longer follow up are still needed to confirm the efficacy and safety of this combination.

Vitreous seeds have three different morphological types; dust seeds, spheres, and clouds. The time for regression of these different types of vitreous seeds was shown to be variable and dependent on the morphology of the seeds (Rishi et al., 2017). Dust seeds responded faster than spheres, while clouds mandate more injections to regress. Other reports have shown that dust seeds usually mandate three injections, spheres mandate six injections, while clouds mandate nine injections before complete seed regression (Francis et al., 2015; Xunda et al., 2016). Our results were similar to these data as the median number of injections mandated to get regression was three for dust seeds, four for spheres, and five for clouds. This is indicative of less volume of active cells in the dust seeds, while clouds harbor collections of aggregated active tumor cells, therefore the injected chemotherapy is unable to get in direct contact with the active tumor cells within the center of the cloud. Thus, more injections are needed to make the cloud fragment initially into dusts, and then these dusts have to be controlled by further injections. This said, although the morphology of seeds affects the number of injections needed to control the tumor, it has no impact on the chances of eye salvage.

Over decades, multiple treatment modalities were adopted to eradicate refractory active seeds before the era of IViC. Intra-arterial chemotherapy with melphalan could control two-thirds of the eyes with active vitreous seeds (Abramson et al., 2012) and this rate of vitreous seeds-control was higher than that with systemic chemotherapy, still, it is not as effective as intravitreal chemotherapy. Furthermore, EBRT was used successfully for control of only 22–64% of eyes with refractory vitreous seeds after the failure of systemic chemotherapy (Berry et al., 2013; Yousef et al., 2020a). Alternately, a combination of IAC and IViC salvaged 87% of the eyes with vitreous seeds (Lee et al., 2016); however, one-half of these eyes had dangerous sight-threatening side effects like hemorrhage, and one-third of them had significant retinal atrophy. We cannot say with full surety if these toxicities are secondary to the general total dose of melphalan that was injected or because of the technique of intra-arterial chemotherapy, which may obstruct the retinal circulation. Nevertheless, intravitreal chemotherapy is potentially more successful than intravenous chemotherapy, IAC, and EBRT for controlling active vitreous seeds in patients with Rb.

In conclusion, our results show that intravitreal melphalan chemotherapy is an effective and relatively safe treatment modality for retinoblastomas and has changed the outcome of eyes with vitreous seeds, significantly improving the ocular oncologists’ capability to salvage eyes. However, there are side effects on both the anterior and posterior segments of the eye, and unanticipated serious toxicity may occur with the standard dose of 20–30 μg melphalan and more so in the eyes that have received multiple treatment modalities.

## Data Availability

The original contributions presented in the study are included in the article/Supplementary Material, further inquiries can be directed to the corresponding authors.

## Abbreviations

Rb, retinoblastoma; KHCC, King Hussein Cancer Center; IViC, intravitreal chemotherapy; IIRC, international intraocular retinoblastoma classification; EBRT, external beam radiation therapy.
